# Brazilian Academy of Neurology: 60 Years

**DOI:** 10.1055/s-0042-1760108

**Published:** 2022-12-29

**Authors:** Ayrton Massaro, Hélio A. G. Teive, Paulo Caramelli

**Affiliations:** 1 Hospital, São Paulo SP, Brazil.; 2 Universidade Federal do Paraná, Hospital de Clínicas, Departamento de Medicina Interna, Serviço de Neurologia, Curitiba PR, Brazil.; 3 Universidade Federal de Minas Gerais, Faculdade de Medicina, Departamento de Clínica Médica, Belo Horizonte MG, Brazil.

The Brazilian Academy of Neurology (ABN – *Academia Brasileira de Neurologia* ) was founded on May 5, 1962 in the city of Rio de Janeiro, under the leadership of Professors Deolindo Augusto de Nunes Couto and Adherbal Tolosa, who were disciples of Professors Antonio Austregésilo (founder of the first school of Neurology in Brazil and first professor of Neurology at the Faculty of Medicine of Rio de Janeiro in 1912) and Enjolras Vampré (founder of the São Paulo School of Neurology in 1925 and first professor of Neurology at the University of São Paulo), respectively. 1 2 The inaugural meeting was held at the Institute of Neurology in Rio de Janeiro, and was chaired by Professor Deolindo Couto, who was is credited as the patron of ABN. 1 2 3 The two masters had the support of 47 other Neurology professors, from different parts of Brazil, many of them direct disciples of these two leaders from the Rio de Janeiro and São Paulo schools of Neurology. 1 2 3

**Figure 1:**
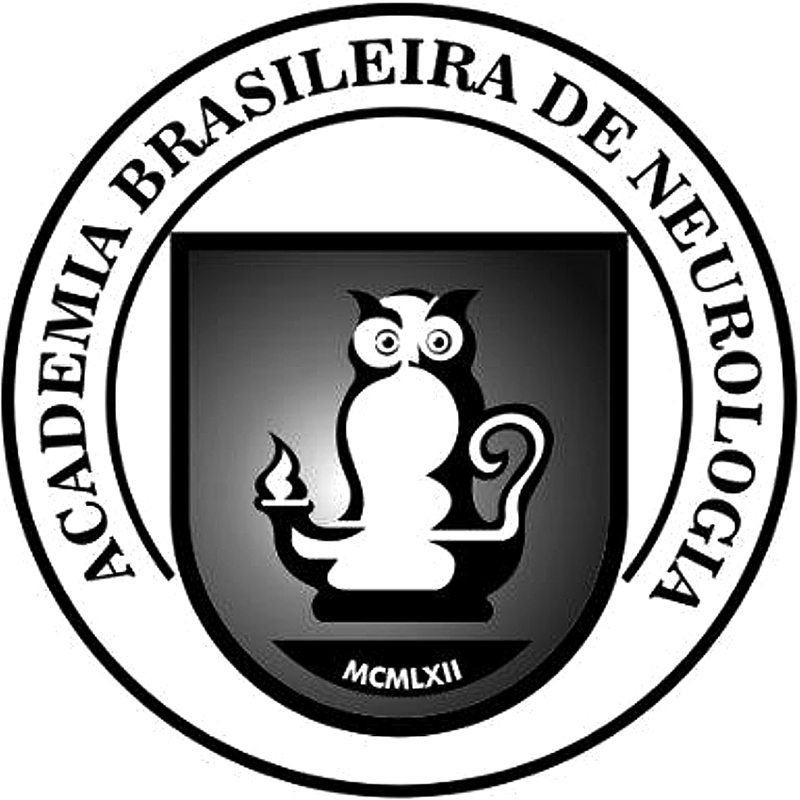
Brazilian Academy of Neurology symbol.

At this time, the ABN symbol was created ( Figure 1 ), which was proposed by Professor Deolindo Couto. It has an owl, associated with a lamp, a square, and a circle, each with a specific meaning. 1 2 The owl, representing an emblem of wisdom, knowledge, clairvoyance and the lamp, symbolizing vigilance, the light that illuminates the night, are surrounded by a white rectangle, which is an ancient symbol of the material world, and that is surrounded by a circle, which represents unity and wholeness. 1 2

The first formal scientific meeting of ABN was held in the city of Curitiba, Paraná, in 1963, from June 30 to July 5, at the rectory of the Federal University of Paraná. 4 Overall, 103 scientific papers were presented, predominantly from the medical schools of São Paulo and Rio de Janeiro. 4 The first official board of ABN included professors Adherbal Tolosa, as president; Paulo Pinto Pupo, as secretary; and Horácio Martins Canelas, as treasurer.

In the same year as its foundation, ABN joined the World Federation of Neurology, and the first exam to obtain the title of specialist in Neurology took place in 1972. 1 2 In this period of 60 years, ABN has achieved exponential growth, with currently more than 5000 active members, distributed in all the states of Brazil. The current board of ABN, with Professor Carlos R. Rieder as president, has greatly modernized the institution, with constant encouragement to create scientific events throughout the nation, culminating in the organization of the Brazilian Congress of Neurology, every two years, which represents one of the main activities of ABN, together with the publication of its official journal, Arquivos de Neuro-Psiquiatria (ANP).

The ANP will complete 80 years of uninterrupted publication in 2023, since its creation, in 1943, by a group of professors from the University of São Paulo, under the initial coordination of Professor Oswaldo Lange and Professor Antonio Spina-França. 5 Recently, the ANP increased its impact factor (JCR), with an exuberant growth, going from a previous index of 1.420 to 2.035, which brought great joy to the entire Brazilian neurological community. 5 This undoubtedly demonstrates the fabulous growth of Brazilian Neurology, as well as the impact of the scientific production published in ANP.

Thus, in its 60 years of existence, ABN has achieved its primary objective, which is to promote the scientific development of Brazilian Neurology.
